# Quantifying the Impact of Linear Regression Model in Deriving Bio-Optical Relationships: The Implications on Ocean Carbon Estimations

**DOI:** 10.3390/s19133032

**Published:** 2019-07-09

**Authors:** Marco Bellacicco, Vincenzo Vellucci, Michele Scardi, Marie Barbieux, Salvatore Marullo, Fabrizio D’Ortenzio

**Affiliations:** 1 Sorbonne Université, CNRS, Laboratoire d’Océanographie de Villefranche, LOV, F-06230 Villefranche-sur-Mer, France; 2 Italian National Agency for New Technologies, Energy and Sustainable Economic Development (ENEA), 00044 Frascati, Italy; 3 Sorbonne Université, CNRS, Institut de la Mer de Villefranche, IMEV, F-06230 Villefranche-sur-Mer, France; 4 Department of Biology, University of Rome “Tor Vergata”, 00133 Rome, Italy

**Keywords:** linear regression methods, bio-optical properties, BGC-Argo, satellite oceanography

## Abstract

Linear regression is widely used in applied sciences and, in particular, in satellite optical oceanography, to relate dependent to independent variables. It is often adopted to establish empirical algorithms based on a finite set of measurements, which are later applied to observations on a larger scale from platforms such as autonomous profiling floats equipped with optical instruments (e.g., Biogeochemical Argo floats; BGC-Argo floats) and satellite ocean colour sensors (e.g., SeaWiFS, VIIRS, OLCI). However, different methods can be applied to a given pair of variables to determine the coefficients of the linear equation fitting the data, which are therefore not unique. In this work, we quantify the impact of the choice of “regression method” (i.e., either type-I or type-II) to derive bio-optical relationships, both from theoretical perspectives and by using specific examples. We have applied usual regression methods to an in situ data set of particulate organic carbon (POC), total chlorophyll-*a* (TChla), optical particulate backscattering coefficient (b_bp_), and 19 years of monthly TChla and b_bp_ ocean colour data. Results of the regression analysis have been used to calculate phytoplankton carbon biomass (C_phyto_) and POC from: i) BGC-Argo float observations; ii) oceanographic cruises, and iii) satellite data. These applications enable highlighting the differences in C_phyto_ and POC estimates relative to the choice of the method. An analysis of the statistical properties of the dataset and a detailed description of the hypothesis of the work drive the selection of the linear regression method.

## 1. Introduction

In technical and scientific applications, the linear regression fit is one of the most common models used to establish a relationship between two variables. Two families of statistical methods, i.e., type-I (ordinary least square, OLS) and type-II (e.g., standard major axis, SMA), were developed to perform a linear regression depending on the properties of the data set [1]. In optical oceanography, the rationale behind the choice of a given method for computing a linear regression fit is often an overlooked question, seldom explained or supported by statistical evidence.

This issue, which also pervades other fields of marine science such as fishery ecology, has already been highlighted by Laws et al. (1981) [2]: “the need to use model II (here type-II) regression methods in many applications have long been recognized, but a glance at the current literature will reveal that most biological oceanographers use model I (here type-I) regression methods exclusively even when model II is clearly needed”. Until the 1980s, the lack of widespread statistical software packages could have been a reason that favoured the application of the most common type-I method. However, as reported in Laws et al. (1981) [2] and Innamorati et al. (1990) [3], the need for a more careful choice of the regression models and methods was already clear in the oceanographic community.

Laws et al. (1981) [2] investigated the problem demonstrating that type-II methods should be applied to in situ data which are affected by instrument and sampling uncertainties. Specifically, they mentioned some common applications for which type-II methods are clearly needed, including, but not limited to, estimation of the phytoplankton chlorophyll-to-carbon ratio from chlorophyll vs. particulate carbon relationship [4,5].

The impact of the choice of regression method on optical oceanographic research is not minor. Linear regression models, in fact, are widely used to predict variables that are difficult or expensive to measure from field measurements. For example, the optical particulate backscattering coefficient (b_bp_) is in situ measured or derived from ocean colour imagery. It is at the base of the estimation of the particulate organic carbon (POC) [6,7,8,9,10] and the phytoplankton carbon biomass (C_phyto_) [11,12,13], both of which are fundamental variables used to constrain and understand the total carbon budget in the ocean [14,15]. Even though there are many works in which regression methods are correctly used and clearly mentioned in the text giving the opportunity to understand and reproduce the work [16,17,18,19,20], there are several cases where no information is provided about the linear regression method used [12,21,22,23,24,25], thus, preventing an evaluation of the impact of the methodology on the derived parameters. The lack of such information is crucial as the use of one method over another can return significantly different estimates on parameters. Indeed, differences due to the application of a sub-optimal regression method, instead, might be considered as errors and propagate if the wrong parameters are then used as inputs for modelling (e.g., empirical algorithms of ocean parameters). For this reason, as McArdle (2003) [26] pointed out “…if the slope, the intercept, or both parameters of the line are important, then care must be taken that the scientific conclusions follow from the data”. In other words, the scientific conclusions must be based on the appropriate methodology, i.e., a methodology adapted to the statistical properties of the data set to be analysed.

In this regard, our primary goal is to evaluate the impact of the linear regression model (and methods) in optical and satellite oceanography. To do so, we quantify the differences between the results obtained applying diverse regression methods to the same bio-optical data set, and investigate the consequences of an inappropriate selection. Namely, we used field measurements collected in the north-western Mediterranean Sea at the BOUSSOLE (BOUee pour l’acquiSition d’une Serie Optique à Long termE) site [27,28,29], during three years of monthly oceanographic cruises (2011–2013) and more than one year (July 2013–November 2014) of Biogeochemical-Argo (aka BGC-Argo) vertical profiles. We applied both type-I and type-II regression methods to determine the coefficients of the linear equations of the total chlorophyll-*a* (TChla)-b_bp_ and b_bp_-POC relationships from discrete samples. Afterwards, we assessed the impact of the derived linear models with to the estimation of C_phyto_ and POC base on the time series of b_bp_ vertical profiles from the BGC-Argo floats and by applying either type-I or type-II regression method. Finally, a similar analysis was also conducted relying on satellite observations, namely C_phyto_ was evaluated, by using 19-years of monthly TChla and b_bp_. 

## 2. Data and Methods

### 2.1. Theoretical Background

Establishing a linear relationship between dependent and independent variables (*y* and *x*, respectively) requires the computation, through linear regression analysis, of the coefficients of a linear equation, i.e., the slope (*B*) and intercept (*A*):

(1) y=B·x+A

The independent variable may be either uncontrolled (i.e., whose variability is affected by random phenomena) or controlled by the investigator, whereas the dependent variable has to be random by definition. The properties of both variables should drive the choice of an appropriate method to perform regression analysis, in contrast to linear correlation analysis, which is only aimed at measuring the strength of the linear relationship between two variables, independent of their properties and of any functional or causal link between them [1].

Although linear fitting is mainly used as a means to highlight a linear relationship between a pair of parameters, often the resulting equation is adopted as a model for further computations or analysis. Thus, the selection of the statistical method used to calculate the slope and the intercept of the linear model is of great importance to minimize the uncertainties associated with the dependent variable.

**Figure A1:**
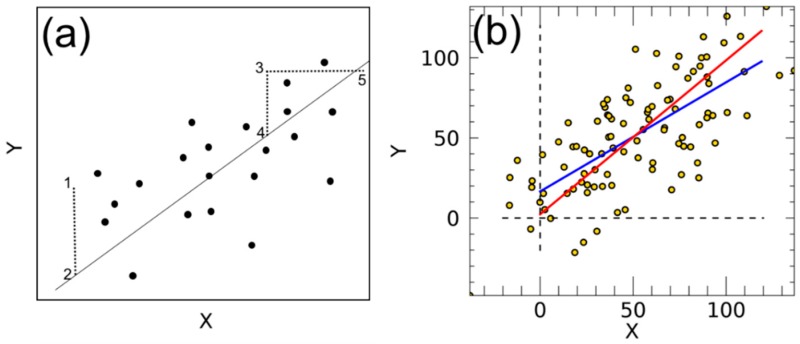
For an OLS line, the error is defined as the vertical dispersion of a point from the straight line (distance 1 to 2) and the quantity minimized is the sum of squares of these linear distances. In case of SMA, on the other hand, the error is defined as the area of the triangle 3-4-5 and the quantity minimized is the sum of these area (redrawn from Smith et al., 2009 [30]) (**a**). Scatter plot and linear fits calculated with OLS (blue) and SMA (red) methods by using a syntehtical datasets with a normal distributed error added to both X and Y variables (**b**).

If the objective of linear regression is the interpolation or extrapolation of a data set, then the most common computational method, OLS, is appropriate independent of the properties of the variables [1,30]. This is the default method that most software use for computing and displaying a fitting line onto a scatter plot and the one which most users are familiar with. Nevertheless, other methods might be more appropriate depending on the goal of the analysis and on the properties of the data set. In such a context, the first step is selecting either a type-I or type-II regression method, which depends on whether the relationship between *x* and *y* variables is symmetric or asymmetric. This means whether or not the variables can be interchanged without altering the hypothesis/assumptions of the work and the derived parameters. An asymmetric relationship underpins a classical linear regression problem whereby the independent variable is characterized by null or low uncertainties as compared to the dependent one. This is the case, for instance, when the independent variable is fully controlled by the investigator or inherently free from uncertainties. A symmetric relationship, on the other hand, occurs when both variables show comparable uncertainties. Asymmetric relationships require type-I regression, whereas symmetric relationships require a type-II regression method [31]. While OLS is the only method to handle type-I problems, several methods can be adopted with a type-II regression. The OLS needs to be used only if the aim of the analysis is to predict the value of the dependent variable (*y*), given the independent one (*x*). This method minimizes the deviations of *y* from the fitting line, i.e., those that are relevant to the prediction of unknown *y* values. Both *x*, *y* and deviations from the fitting line are instead relevant if the goal of the regression analysis is to assess the slope and/or intercept of the best regression line (see Figure A1 in Appendix A). In this case, OLS is not the most appropriate while type-II methods have to be followed. These type-II methods provide slope and intercept estimates that are, in most cases, significantly different from those obtained through OLS. Type-II methods are: major axis (MA), standard major axis (SMA) and ranged major axis (RMA). Note that the latter acronym is also used for reduced major axis, which is a synonym for SMA [31].

According to [1], MA is the appropriate method when: 1) data distribution is bivariate normal; 2) *x* and *y* variables are dimensionless or share the same units, and 3) the error variance is of similar magnitude for the two variables. RMA can handle variables whose units are heterogeneous because data is normalized before computing a MA. Because of this normalization, possible outliers have to be identified and eliminated from the data set, otherwise they could significantly alter the results. In the SMA, the slope is calculated as the ratio of the standard deviation of *y* to that of *x* [1]. However, SMA has two drawbacks: it should be computed only when the correlation between *x* and *y* and is significant, and its slope cannot be tested for significance.

Differences estimates of the slopes and intercepts obtained by applying different methods depend on the degree of correlation between *x* and *y*. When *x* and *y* are strongly significantly correlated the differences between the three type-II methods based on the major axis are usually small. Nonetheless, all of them differ from OLS regression to a larger extent [1]. Yet, when the correlation coefficient tends to 1, the differences between type-II methods and OLS diminish respectively (see Appendix A). 

In the following sections, we are concentrating only on SMA, as a type-II method, and OLS, as type-I, because both are the most widely used in optical oceanography and field sciences (for more details about their mathematical treatment see Appendix A). Furthermore, MA and RMA cannot be applied to our dataset (i.e., heterogeneous parameters with different units, presence of outliers) [1,26,30,31].

### 2.2. Field and Satellite Measurements

#### 2.2.1. Cruise Data

**Figure 1:**
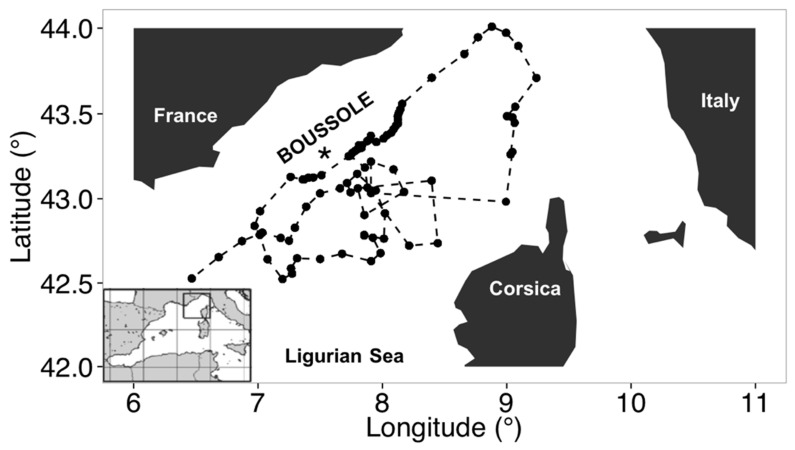
The northwestern Mediterranean Sea showing the southern coast of France, the island of Corsica, and the location of the BOUSSOLE buoy in the Ligurian Sea (black star) redrawn from [22]. Black dots are the locations where the float surfaced, while the float trajectory is overlaid in the plot with dashed black line.

The BOUSSOLE project started in 2001, and its activities are developed around a bio-optical buoy located in the deep waters (2440 m) of the Ligurian Sea, one of the sub-basins of the Western Mediterranean Sea [27,28,29] (http://www.obs-vlfr.fr/Boussole/html/home/home.php). Figure 1 shows the area of study and the location of BOUSSOLE site. The BOUSSOLE site has been visited monthly for buoy servicing, during which 0–400 m casts are performed for the acquisition of conductivity, temperature and pressure (SBE 911plus, SeaBird Inc., Bellevue, WA, USA). Likewise, water samples are collected at 12 depths (400, 200, 150, 80, 70, 60, 50, 40, 30, 20, 10 and 5 m) with 12 L Niskin bottles mounted on a General Oceanic Rosette equipped with an SBE 32 Carousel Water Sampler, and then subsampled into polycarbonate bottles. An independent optical package is coupled to the conductivity, temperature and density (CTD)/Rosette for the acquisition of inherent optical properties. In this study, we used measurements of POC, TChla and b_bp_ collected from October 2011 to December 2013, whose measurement protocols are summarized below:

Particulate organic carbon. Water is filtered through Whatman 25 mm GF/F glass-fiber filters (filtered volume from 2.27 to 5.5 L, depending on samples). Filters were washed beforehand using the soxhlet extraction method with dichloromethane. After filtration, samples were put into petri dishes and stored in liquid nitrogen for the duration of the cruise and then transferred into −80 °C freezer until analysis, which took place within 12 months from sampling. Two days before the analysis, the filters were stored in a drying chamber at 50 °C during 1 night and then decarbonated with HCl solution. Finally, the filters were analyzed using a carbon, hydrogen and nitrogen (CHN) analyzer (Perkin Elmer 2400 series II) with the combustion analysis method. The relative uncertainty of the POC was estimated at < 1% from inter-calibration exercises of the analytical platform used here with other French laboratories (L. Coppola personal communication).

Total chlorophyll-*a*. Samples for the determination of phytoplankton pigments were filtered through 25 mm Whatman GF/F (0.7 µm retention capacity), put into petri dishes and stored in liquid nitrogen for the duration of the cruise. They were then transferred into −80 °C freezer until the analysis, which took place within 6–8 months from sampling. Pigments were identified and quantified with the high-performance liquid chromatography (HPLC) technique following [32]. The total chlorophyll-*a* concentration is computed as the sum of the concentrations of chlorophyll-*a*, chlorophyllide-*a*, and divinyl chlorophyll-*a*. Uncertainties in the methodology of the analytical platform used here were evaluated in a series of Round-Robin experiments (SeaHARRE-1 to 5; https://oceancolor.gsfc.nasa.gov/docs/technical/), and is of 6% for TChla used here (report of the analyses by J. Ras and M. Ouhssain).

Particulate backscattering coefficient. The total volume scattering function at 140°, β(140, λ), was measured with a HydroScat-VI backscattering meter (HOBI Labs) at 6 wavelengths (420, 442, 488, 550, 620, 700 nm). The instrument was deployed within an independent Inherent Optical Properties (IOPs) package mounted below the CTD/Rosette, with the optics field of view looking at nadir. In this study, the down-cast was used to insure the medium was not perturbed during the measurement (i.e., to avoid possible disaggregation of particulate). Thus, the measurements had a maximum time lag of approximately 30 min with the discrete POC and HPLC sampled during the up-cast. The spectral particulate backscattering coefficient, b_bp_(λ), is obtained following [33], with few differences: (1) one dark 0–50 m β(140, λ) profile was measured, averaged and subtracted from all profiles within each cruise; (2) data were binned around ±0.5 m of each nominal depth (1 m resolution); (3) the total absorption and beam attenuation coefficients used for the σ correction [34] were measured respectively with an *a*-Sphere absorption meter (HOBI Labs) and a with Gamma-4 transmissometer (Hobi Labs).

#### 2.2.2. BGC-Argo Floats Data

As part of this study, we used data obtained from two of BGC-Argo profiling floats deployed in the North Western Mediterranean Sea for a total 87 vertical profiles as displayed in Figure 1. The float referenced as WMO (World Meteorological Organization) N°6901496 was deployed in the Ligurian Sea near the BOUSSOLE buoy on 15 July 2013. In March, due to a significant bio-fouling of the optical sensors, this float was recovered, cleaned and deployed on again the 14 March 2014 under the reference of WMO N°6901776. Thus, the two BGC-Argo time series were joined end to end, corresponding to upward casts collected between 16 July 2013 and 25 October 2014.

All casts started from the parking depth at 1000 m at a time that was sufficient for surfacing around noon (local time). Vertical resolution of acquisition was 10 m between 1000 m and 250 m, 1 m between 250 m and 10 m, and 0.2 m between 10 m and the surface. Here, only “noon” casts were used. Following procedures described in [35], the b_bp_(700) profiles were calibrated, quality-controlled and additionally corrected by removing positive spikes greater than twice the 90th quantiles of the residual signal calculated as the difference between the profile and a median filter (window of 5 dots).

#### 2.2.3. Ocean Colour Data

The full ESA OC-CCI v3.0 (European Space Agency Ocean Colour-Climate Change Initiative version 3.0) monthly TChla (mg m^−3^) and b_bp_ (m^−1^; 443nm) data time-series at 4 km resolution for the period 1997–2015 over the global ocean was downloaded from the ESA-CCI website (http://www.esa-oceancolour-cci.org/). ESA-CCI products are the results of the merging between SeaWiFS, MERIS, MODIS-Aqua, and VIIRS time-series [25,36,37,38]. TChla was estimated with a blending of the OCI (as implemented by NASA, itself a combination of CI and OC4), the OC5 (NASA, 2010) and the OC3 algorithms (http://www.esa-oceancolour-cci.org/?q=webfm_send/684). The Quasi-Analytical Algorithm (QAA) was used to compute b_bp_ [39,40]. The accuracy of the QAA algorithm was demonstrated in several recent studies [12,13,19,23,24,25,41]. Both datasets were remapped at 100 km resolution, enough to resolve the broader oceanographic scales of variability. In such a context, monthly TChla and b_bp_ data were selected for the specific area of the northwestern Mediterranean basin to maintain the same domain of the analysis performed by using field measurements (see Figure 1).

### 2.3. Statistics

In such a context, the following statistical indicators have been used to quantify the impact of regression methods in deriving bio-optical relationships and subsequent biogeochemical parameters (i.e., C_phyto_ and POC):

(i) “*Anomalies*” here defined as the difference between parameters established with OLS and SMA linear regression methods.
(ii) The relative percentage differences (RPD) between parameters computed by the application of the unsuitable and suitable method.

## 3. Results and Discussion

In the following, we use models of C_phyto_ as a function of the TChla -b_bp_ relationship, and POC as a function of b_bp_ as examples to highlight the impact of using a regression method not adapted to the data set on typical bio-optical oceanographic problems.

### 3.1. Total Chlorophyll-a versus Optical Backscattering 

Behrenfeld et al. (2005) [11] proposed the estimation of C_phyto_ based on the relationship between TChla and b_bp_(443) and applied their model to SeaWiFS ocean color data on a global scale. Bellacicco et al. (2016) [12] revisited the model for regional tuning respective to the Mediterranean Sea and used the 555 nm band instead of 443 nm for b_bp_. Recently, Bellacicco et al. (2018) [13] generalized this approach on a global scale by using b_bp_(443). The equation for the computation of C_phyto_ is:

(2) $$C_{\mathrm{phyto}}=\left[b_{\mathrm{bp}}\left(\lambda \right)-b_{\mathrm{bp}}^{k}\left(\lambda \right)\right]\cdot \mathrm{SF}$$

where λ is the wavelength. Here, we used b_bp_ at 700 nm for compatibility also with BGC-Argo float measurements. The b^k^_bp_(700) is the backscattering coefficient, at 700 nm, of the background fraction of non-algal particles that does not covary with TChla (e.g., heterotrophic bacteria and viruses) [11]. This value corresponds to the b_bp_(700) when TChla is zero: it is the intercept of the linear fit between the two variables. SF is the scaling factor chosen to give satellite Chl:C values (average value of 0.010) consistent with laboratory results, and also for the average contribution of phytoplankton to total particulate organic carbon (±30%) to be consistent with field estimates. In the original work, SF is equal to 13,000 mg C m^−2^ [11]. Here, taking into account the change of wavelength for b_bp_(700 nm instead of 443 nm) and to remain consistent with [11], we computed, according to in situ data, a SF of 16,455 mg C m^−2^, 26% more with respect to the value of the original work. About the b^k^_bp_(700), Bellacicco et al. (2016) [12] demonstrated that b^k^_bp_ (555) varies both in space and time. However, for sake of simplicity, we considered it to be a constant as in the original work of Behrenfeld et al. (2005) [11]. The main assumption of the model is the good relationship between TChla and b_bp_ [12,13,23]. The first order co-variability between TChla and b_bp_ is expected because phytoplankton cells contain TChla and also act as light backscatterers [13,42,43,44]. This co-variability also indicates that particles population abundance covaries with phytoplankton biomass, whereas the physiological photoacclimation process plays a secondary role in determining the chlorophyll variations [11,44]. In such a specific context, the underlying hypothesis is that TChla is the independent variable while b_bp_ is the dependent one. There is no likelihood of interchanging the variables for the evaluation of the b^k^_bp_. Indeed, b^k^_bp_ is defined as the intercept of the linear regression fit between TChla and b_bp_, and it is the b_bp_ when TChla is equal to 0. The choice of the most appropriate regression method is founded upon which is the dependent variable and which is the independent one. In this case, the main goal is the estimation of a parameter (b^k^_bp_), allowing for the definition of another parameter (C_phyto_), thus OLS is the preferable method to be applied [11,12,13]. 

**Figure 2:**
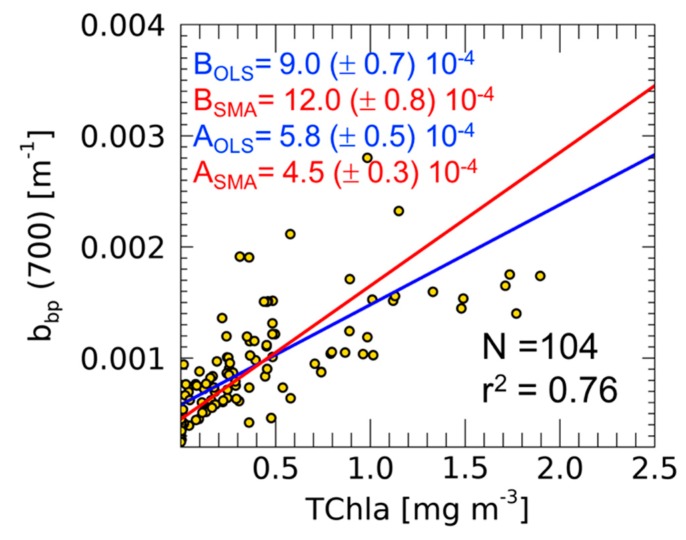
Scatter-plot and linear fit (continuous lines) calculated with ordinary least square (OLS) (blue) and standard major axis (SMA) (red) methods in the TChla-b_bp_ relationship at the BOUSSOLE site. For both the coefficients, intercepts (A) and slopes (B), the standard errors are also indicated.

The use of either OLS or SMA has consequences on the final C_phyto_ biomass estimates that we can compute using in situ data. Furthermore, the intercept of the linear fit (i.e., b^k^_bp_ coefficient), in fact, has a biological meaning, as being the background contribution of the non-algal particles to the total b_bp_ [11]. Figure 2 shows the TChla-b_bp_ relationship with indicated both slopes and intercepts as computed by applying the two different regression methods. Here, the intercept varies from 5.8 (±0.5) × 10^−4^ to 4.5 (±0.3) × 10^−4^ m^−1^ when calculated with OLS and SMA, respectively (Figure 2). These values are lower than those reported for the same region (though only surface measurements were used) [12]. This is consistent with a theoretically higher carbon (and its proxy b_bp_) to TChla ratio in more illuminated waters [14].

As discussed before, the definition of cause (independent variable) and effect (dependent variable) is thus fundamental and represents the working hypothesis. If one focuses on the relationship between TChla and b_bp_, the goal being their comparison, the SMA (or RMA) has to be applied because it is statistically more robust in the context of the analysis of field measurements as explained in Section 2.1.

To further underline the difference in results when applying OLS or SMA methods, we computed the total C_phyto_ from Equation (2) and by using the b_bp_(700) 0–400 m profiles collected during the BOUSSOLE cruises. To each profile, we applied the b^k^_bp_(700) values (i.e., intercepts) obtained after the application of both OLS and SMA methods (Figure 2). The RPD on C_phyto_ estimation is equal to 23.5% (overestimation of total C_phyto_ using SMA instead of the appropriate OLS method).

Additionally, in order to evaluate how the estimate of C_phyto_ changes when using either methods, we applied the relationships reported in Figure 2 to the time series of b_bp_(700) vertical profiles from the BGC-Argo dataset. When assessing the integral of C_phyto_ over depth and time, the RPD between C_phyto,SMA_ and C_phyto,OLS_ is 28.7%. In this example, the use of SMA (the less adapted method) leads to an overestimation of C_phyto_.

**Figure 3:**
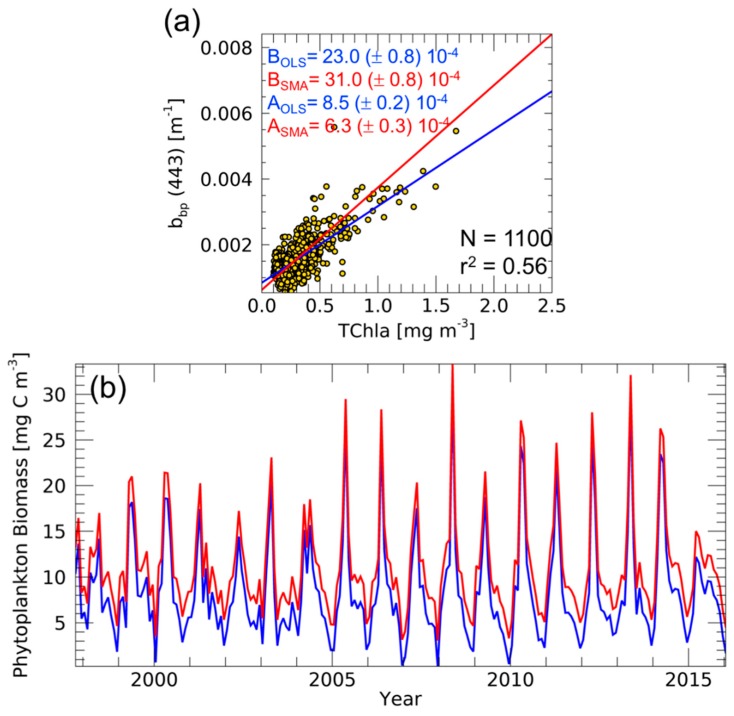
Scatter plot between TChla and b_bp_ from ocean colour data in the northwestern Mediterranean Sea with linear fits (continuous lines) calculated with OLS (blue) and SMA (red) methods (**a**). For both the coefficients, intercepts (A) and slopes (B), the standard errors are also indicated. Time series of C_phyto_ (**b**) based on the b^k^_bp_ computed by OLS (in blue) and SMA (in red) methods.

Furthermore, we conducted the analysis by using 19-years of monthly ocean colour data of TChla and b_bp_(443) for the period 1997–2015 as shown in Figure 3. As described earlier, the good relationship between TChla and b_bp_ enables defining the b^k^_bp_ coefficient, a fundamental parameter for the C_phyto_ computation. Figure 3a shows a moderate correlation between TChla and b_bp_ (r^2^ equal to 0.56) in the northwestern Mediterranean Sea. The correlation implies the reliable estimation of b^k^_bp_ coefficient by using all the pixels for the period 1997–2015 [12,13]. In such a context, the b^k^_bp_ is 8.5 (±0.2) × 10^−4^ m^−1^ (with the OLS method), a value consistent with the order of magnitude found by a recent work always based on ocean colour data [13]. With the SMA, the b^k^_bp_ becomes lower with respect to the computation performed by OLS: 6.3 (±0.3) × 10^−4^ m^−1^. Figure 3b shows the subsequent crucial application of this coefficient on the satellite averaged b_bp_ time series for the C_phyto_ computation by using Equation (2) (443 nm instead of 700 nm as a wavelength of reference). Figure 3b shows how both the obtained C_phyto_ time series follow a similar temporal pattern but with different values. Regarding the entire time series, the mean difference between C_phyto,SMA_ (with SMA-based b^k^_bp_) and C_phyto,OLS_ (with OLS-based b^k^_bp_) is 2.56 mg C m^−3^, that is the 28% of the mean C_phyto,OLS_: C_phyto,SMA_ overestimates C_phyto,OLS_. Therefore, in this specific context, there is a general overestimation of C_phyto_ if one uses the SMA method instead of OLS. This critical point has to be taken into account because of its potential impact in the case of phytoplankton carbon studies on regional and global scales, mostly in ocean carbon budget studies.

### 3.2. Optical Backscattering vs. Particulate Organic Carbon

The POC is often linearly related to b_bp_ [6,7,8,9,10,23] as follows:

(3) $$\mathrm{POC}=B\cdot b_{\mathrm{bp}}\left(\lambda \right)+A$$

where B is the slope and A is the intercept of the linear regression fit following the general Equation (1). As suggested by Loisel et al., (2001) [6], sub-micrometer particles are efficient backscatterers, such that there is confirmation that the dominant contribution to particulate organic carbon in the ocean is due to sub-micrometer particles that are in sufficiently higher concentrations allowing for dominance of the b_bp_ in oceanic water determining, therefore causing a strong correlation with POC.

The high correlation is caused by the dominance of organic particle concentration in controlling changes in both POC and b_bp_. In such a context, it is possible to interchange POC and b_bp_ for a simple comparison aimed at establishing a relationship between them, i.e., not for optimizing slope and intercept in view of a further application.

If the principal goal is to estimate POC, the appropriate method to be used is type-II regression, owing to methodological uncertainties in both measurements that have to be accounted for. 

**Figure 4:**
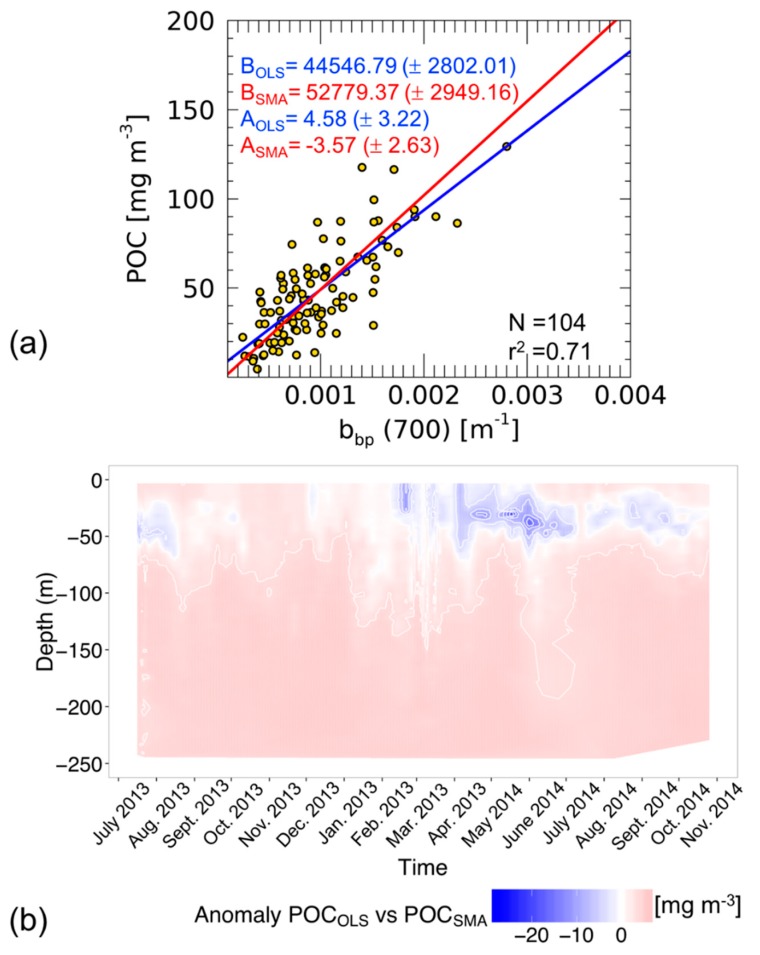
Scatter-plot and linear fits calculated with OLS (blue) and SMA (red) methods in the b_bp_-POC relationship at the BOUSSOLE site (**a**). For both the coefficients, intercepts (A) and slopes (B), the standar errors are also indicated. Time series anomalies of particulate organic carbon (POC) derived from BGC-Argo b_bp_ vertical profiles (0–250 m) using OLS and SMA and relationships (**b**).

Several works reported results of a linear regression between POC and b_bp_ from both satellite and in situ data (Table 2 in Thomalla et al., 2017 [10]). Figure 4 shows the established linear relationship between POC and b_bp_(700) using both SMA and OLS methods. Our estimates of the slope and the intercept, computed using both methods, are consistent with previous results from the Mediterranean Sea [6], Atlantic and Pacific Oceans [7], Southern Ocean [10] and North Atlantic Ocean [23].

Figure 4a shows that the slope and intercept computed using OLS are significantly lower than that computed with SMA (with a higher intercept point). This is a good example of the extent to which the choice of a regression method affects the estimate of the coefficients of the linear fit. As previously presented, these properties depend on minimizing y deviations only, for OLS, or the combination of x and y deviations for SMA. In the case of SMA, reduced uncertainties on the independent variable might balance higher uncertainties on the dependent variable, thus optimizing the overall agreement between the regression line and the data points. It is worth noting that the determination coefficient (r^2^, i.e., the variance explained by the linear model) as well as the correlation coefficient (r, i.e., a measure of the linear correlation between two variables) are not dependent on the regression method.

To quantify how the estimate of POC changes when using OLS or SMA, we have applied the relationships reported in Figure 4a to a time series of b_bp_(700) vertical profiles acquired from BGC-Argo floats in the same area sampled to establish the linear models. Figure 4b shows the anomalies of POC as the difference between POC estimated using linear models based on OLS and SMA methods (POC_OLS_ and POC_SMA_ respectively). The anomalies, in general, are weak; however, several areas of large differences have impacted the computation of the POC budget over the time series. In detail, at the end of spring 2014 the largest anomalies are between 5.0 and 20.0 mg m^−3^ in surface waters. In other periods, the anomalies are between −10.0 and +5.0 mg m^−3^. When evaluating the integral of POC along the water column and over time, the RPD between POC_OLS_ and POC_SMA_ is 13.3%, showing the importance of selecting the correct regression method which avoids an incorrect estimate of the POC budget. In this example, the use of OLS (the unsuitable method) causes an overestimation of POC.

In both of the examples analyzed here (i.e., Figure 2 and Figure 4), the differences in slope and intercept estimates obtained from OLS and SMA methods are quite substantial. These differences could be even greater for data sets with a lower correlation between the two variables [2]. This highlights that the selection of a statistical method not fitted to the data set may introduce substantial errors when the derived linear model is used to estimate the dependent variable from a direct or indirect measurement of the independent variable. The same effect is also evident in the empirical algorithms applied to satellite ocean colour imagery [6,8] or on BCG-Argo vertical profiles [10], which therefore should be assessed by applying the appropriate linear regression method.

## 4. Conclusions

In this study, we have used both type-I (i.e., OLS) and type-II (i.e., SMA) methods with bio-optical data collected over three years of monthly oceanographic cruises at the BOUSSOLE site (Figure 1) to derive linear regression coefficients (i.e., slopes and intercepts) that were then applied to BGC-Argo vertical profiles for the estimations of phytoplankton carbon biomass and particulate organic carbon. In addition, a specific analysis using ocean colour data is addressed. The main goal is to quantify the impact of the linear regression methods in satellite optical oceanography. Our analysis has shown that:

- The phytoplankton carbon biomass based on the TChla-b_bp_ relationship needs to be computed using the OLS method due to the asymmetry assumption between the two variables. In such a context, the intercept of the linear fit between TChla and b_bp_, which is necessary to compute the C_phyto_, represents the fraction of b_bp_ that does not co-vary with TChla, confirming that the dependent and independent parameters cannot be interchanged from a theoretical perspective. Only in this specific case, the application of the SMA is unsuitable, as it assumes symmetry of the parameters. Its application always determines an overestimation of phytoplankton carbon biomass.
- For all linear regression analysis in which the main aim is to compare two parameters (e.g., b_bp_-POC or TChla-b_bp_), the most appropriate method is SMA due to its theoretical symmetry, and because of the uncertainties that affect both variables. It is thus possible to interchange the *x* and *y* axes without any impact on the interpretation of the results.

The main outcome of these examples is that the choice of method to determine the coefficients of the linear model significantly impacts C_phyto_ and POC retrievals. The introduction of sizeable errors is a key factor in the carbon budget estimates when linear models are used on a global scale. Indeed, the total C_phyto_:POC ratio utilizing the time series of b_bp_(700) vertical profiles give an RPD of 13.6% overestimation using C_phyto,SMA_ to POC_OLS_ (the ratio computed using both unsuitable approaches) with respect to the ratio C_phyto,OLS_ to POC_SMA_ (the appropriate methods to be used). It has to be kept in mind that two single relationships are applied to the full time-series of the BGC-Argo floats in the example shown here. It is understood however that spatio-temporal variations of the two relationships exist and could have an impact on the budget estimates. In this work, we thus highlighted the importance of the selection and use of the correct regression method. The choice of the model, and hence the method, has to be done a priori relative to any computation based on the data set properties. Given that, it cannot be overlooked that a fraction of the variation of the data around a linear regression fit can also be due to biogeochemical variability rather than error measurements. This type of error represents that portion of variability unresolved by the fitting function adopted, especially in case of ocean color data, where retrieval models are ofter oversimplified. Furthermore, in case of high correlation between variables, both slope and intercept estimations computed by type-I and type-II regression methods do not show large differences between. Therefore, for a correct application of linear regression methods in optical and satellite oceanography, a deeper study of the relationship between the two variables from a theoretical point of view needs to be performed. In fact, as demonstrated, the influence of the unsuitable method in cases of carbon estimations can be considerable and potentially impactful in the context of global carbon budget studies from space or by using field measurements.

## Appendix A. Mathematical Details

In this section, the differences between OLS and SMA methods are described with mathematical detail and from a theoretical point of view.

The linear regression is used to compute the parameters of a first-degree equation relating variables y and x. The equation for a simple linear regression is defined as:

(A1) $$\hat{y}=B\cdot x+A$$

This corresponds to the equation of a straight line that fitting a cloud of points, on a Cartesian coordinate system, in some optimal way, and establish a model to predict $\hat{y}$ for any value of x. *A* is the estimate of the intercept of the regression line with the *y* axis and *B* is the slope of the regression line or regression coefficient. When using this type of regression, one must be aware of the fact that a linear model is imposed on the dataset. In other words, one assumes that the relationship between the two variables may be well described by a straight line and that the vertical dispersion of observed values above and below the line is the result of a random process. The difference between the observed and estimated values along y is:

(A2) $$\varepsilon_{i}=\left(y_{i}-\hat{y}\right)$$

for each observation *i* and may be positive or negative. The term $\varepsilon_{i}$ is called the residual value of observation $y_{i}$ after fitting the regression line. If we include the $\varepsilon_{i}$ term in the Equation (A1), it allows one to describe exactly the ordinate value $y_{i}$ of each point ($x_{i}$, $y_{i}$) of the dataset; $y_{i}$ is equal to the value $\hat{y_{i}}$ , as predicted by the regression equation plus the residual $\varepsilon_{i}$, as follows:

(A3) $$y_{i}={\hat{y}}_{i}+\varepsilon_{i}=B\cdot x_{i}+A+\varepsilon_{i}$$

This equation is the linear model of the relationship. The term ${\hat{y}}_{i}$ is the predicted or fitted value corresponding to each observation *i*. The model assumes that deviations from the linear relationship are only on the vertical axis (i.e., “errors” $\varepsilon_{i}$ are only associated to the response variable $y_{i}$; “errors” associated with the estimation of x, $\delta_{i}$, are equal to zero). The term “error” is the term traditionally used to indicate the deviations of all kinds due to random processes, and including those linked to measurements or methodology.

In simple linear regression, one is looking for the straight line with equation $\hat{y}=B\cdot x+A$ that minimizes the sum of square of the vertical residuals $\varepsilon_{i}$, between the observed values and the regression line (see Figure A1a). This is the principle of the least squares method. This sum of square residuals, $\Sigma {\left(y_{i}-\hat{y_{i}}\right)}^{2}$ (as in case of the OLS method), offers the advantage of providing a unique solution, which would not be the case if one chose to minimize, for example $\Sigma \left|y_{i}-\hat{y_{i}}\right|$. On the other hand, the SMA method minimizes the sum of the product of the x and y deviations, $\Sigma \left(x_{i}-\hat{x_{i}}\right)\;\left(y_{i}-\hat{y_{i}}\right)$ which is the equivalent to the area of triangles formed by the deviations of a point from the line in the x and y directions (see Figure A1a). Figure A1b shows how the slopes and intercepts change after the application of OLS and SMA methods.

The solution for Equation (A1), satisfying the least square criterion can be found using partial derivatives and is:

(A4) $$B_{OLS}=\frac{s_{xy}}{s_{x}^{2}};\;A=\bar{y}-B_{OLS}\cdot \bar{x}$$

in which $s_{xy}$ and $s_{x}^{2}$ are estimates of the covariances and variance, respectively.

In case of SMA, or reduce major axis (e.g., a specific case of type-II model), the slope is computed as:

(A5) $$B_{SMA}=\sqrt{\frac{s_{y}^{2}}{s_{x}^{2}}}=\pm \left(\frac{s_{y}}{s_{x}}\right)$$

This formula is obtained from the classical and more general formula:

(A6) $$B=\frac{s_{y}^{2}-\theta s_{x}^{2}+\sqrt{{\left(s_{y}^{2}-\theta s_{x}^{2}\right)}^{2}+4\theta s_{xy}^{2}}}{2s_{xy}}$$

where $s_{y}^{2}$ and $s_{x}^{2}$ are the estimated variances of y and x, respectively, $s_{xy}$ is their covariance and θ is the ratio between the variances of the two errors terms, $\frac{\sigma_{\varepsilon }^{2}}{\sigma_{\delta }^{2}}$. The formula of the slope of the SMA method is a specific case of the more general Equation (A6). In the SMA slope computation, it is assumed that the error variances $\sigma_{\varepsilon }^{2}$ and $\sigma_{\delta }^{2}$ of y and x, respectively, are proportional to their respective variances $\sigma_{x}^{2}$ and $\sigma_{y}^{2}$. This means that:

(A7) $$\frac{\sigma_{\varepsilon }^{2}}{\sigma_{y}^{2}}=\frac{\sigma_{\delta }^{2}}{\sigma_{x}^{2}}$$

Replacing the variances, $\sigma_{\varepsilon }^{2}$ and $\sigma_{\delta }^{2}$, with their unbiased estimates, $s_{y}^{2}$ and $s_{x}^{2}$, gives a value of θ equal to $\frac{s_{y}^{2}}{s_{x}^{2}}$.

Since the square root $\sqrt{\frac{s_{y}^{2}}{s_{x}^{2}}}$ can be positive or negative, the sign of the slope estimate is given by the sign of the Pearson correlation coefficient (*r*) which is the same as that of the covariance $s_{xy}$ in the denominator of the Equation (A7) or numerator of Equation (A5). The $B_{SMA}$ is also the geometric mean of the OLS regression coefficient of y on x, thus it can be also defined as:

(A8) $$B_{SMA}=\frac{B_{OLS}}{r_{xy}}\;when\;r_{xy}\neq 0$$

Therefore, one can compute $B_{SMA}$ using the value of $B_{OLS}$ and $r_{xy}$ provided by an OLS regression algorithm. This equation also shows that when the variables are highly correlated (*r* tends to 1), $B_{SMA}$ tends to $B_{OLS}$. When they are not, $B_{SMA}$ is always larger than $B_{OLS}$ for positive values of *r*, and smaller for negative values of *r,* in other words, $B_{OLS}$ is always closer to 0 than $B_{SMA}$. For additional details about the theory, mathematical features and a deeper description of regression models and methods, see also [1,26,30,31].
